# Reduced risk for placental malaria in iron deficient women

**DOI:** 10.1186/1475-2875-10-47

**Published:** 2011-02-23

**Authors:** Edward L Senga, Gregory Harper, Gibby Koshy, Peter N Kazembe, Bernard J Brabin

**Affiliations:** 1 University of Malawi College of Medicine, Biochemistry Department, Blantyre, Malawi; 2 Child and Reproductive Health Group, Liverpool School of Tropical Medicine, Liverpool, UK; 3 Emma Kinderziekenhuis, Academic Medical Centre, University of Amsterdam, the Netherlands; 4 Department of Community Child Health, Royal Liverpool Children's Hospital NHS Trust Alder Hey, Liverpool, UK; 5 Baylor College of Medicine Children's Foundation, Lilongwe, Malawi

## Abstract

**Background:**

Nutritional iron deficiency may limit iron availability to the malaria parasite reducing infection risk, and/or impair host immunity thereby increasing this risk. In pregnant women, there is evidence of an adverse effect with iron supplementation, but the few reported studies are strongly confounded.

**Methods:**

A case control study in pregnant Malawian women was undertaken in Chikhwawa southern Malawi in order to describe iron status in relation to placental malaria controlling for several confounding factors. Pregnancy characteristics were obtained and a blood sample at delivery. A full blood count was performed and serum ferritin and transferrin receptor quantified by enzyme-linked immunoassay. DNA analysis was used to identify genetic polymorphisms for ABO phenotype, hemoglobin HbS, and glucose -6 phosphate dehydrogenase deficiency. Placental tissue was obtained and malaria histology classified as active, past or no malaria infection.

**Results:**

112 cases with placental malaria were identified and 110 women with no evidence of placental infection. Iron deficiency was less frequent in women with placental *Plasmodium falciparum *infection. In those with acute, chronic or past placental infections the odds ratio for iron deficiency was 0.4, 95% CI 0.2-0.8, p = 0.01; for acute and chronic infections 0.4, 0.2-0.8, p = 0.006; for acute infection 0.3, 0.1-0.7, p = 0.001. The association was greater in multigravidae.

**Conclusion:**

Women with either acute, or acute and chronic placental malaria were less likely to have iron deficiency than women without placental malaria infection There is a priority to establish if reversing iron deficiency through iron supplementation programs either prior to or during pregnancy enhances malaria risk.

## Background

The influence of iron deficiency on infection risk has been a long-standing clinical concern [1], related to the concept that restriction of availability of iron for growth of pathogens will inhibit pathogen proliferation, and conversely making iron available would increase infection risk [2]. A recent Cochrane analysis of 68 trials of iron supplementation observed an increased risk of malaria with iron in trials that did not provide malaria surveillance and treatment [3]. The Cochrane review did not include studies of pregnant women, yet there are 85.3 million pregnancies in areas with *Plasmodium falciparum *transmission, 54.7 million of which occur in areas with stable transmission and 30.6 million in areas with unstable transmission [4].

Iron-malaria interactions in young non-pregnant women also have been little studied and it is unknown whether improving iron status of young women prior to pregnancy living in malaria endemic areas would increase their risk for pregnancy related malaria. Weekly iron and folic acid supplementation for all women of child-bearing age also has been recommended by the World Health Organization [5], although the safety of this strategy in terms of malaria risk has not been assessed.

Only five published studies on the effects of iron treatments or status during pregnancy on the prevalence of maternal malaria have been identified[6-10]. One showed an association of recent haematinic use with increased risk of *Plasmodium vivax *infection which was not related to parity [6], another no association of haematinics with placental *P. falciparum *infection in multigravidae with HbAS genotype [7]. Two uncontrolled studies reported parenteral iron given to severely anaemic women was associated with higher risk of *P. falciparum *malaria at delivery [8,9], one of which reported the association in primiparae, but not multiparae [9] As these women receiving parenteral iron all had severe antenatal anaemia, they would be the more likely to have malaria at the time of treatment and delivery independent of iron prescription. A single cross-sectional study at delivery reported iron deficiency was associated with decreased the risk of placental parasitaemia especially in the first pregnancy [10].

Multigravidae have reduced prevalence of *P. falciparum *infection, which is attributed to parity specific immunity to malaria [11], although as they have higher risk of iron deficiency due to the cumulative iron needs of successive pregnancies, then their iron status could influence their immunity to malaria. Other factors, including ABO blood group phenotype [12] and adolescence, are also important for defining malaria risks in pregnancy [13]. The rationale for the present study was to determine the association of iron deficiency at delivery with placental malaria controlling for these factors, as well as using a histological classification of placental malaria which characterizes acute, chronic, and past placental infections.

## Methods

### Study sample

This was a case control study conducted between January and July 2005 at Montfort Hospital, in Southern Malawi amongst all pregnant women who attended the hospital for delivery and who provided informed consent. Malaria was highly endemic in this area and transmission occurred year round but intensifies during the rainy season from April to December. A total of 647 women were enrolled who attended the hospital for delivery. Women were approached during the first stage of labour and informed consent was requested for participation. A questionnaire was completed by a trained midwife requesting information on age, parity, literacy, obstetric history, bed net and anti-malarial use. Women with emergency obstetric problems (toxaemia, haemorrhage, obstructed labour or sepsis), or with recent blood transfusion were excluded. Maternal mid-upper-arm-circumference (MUAC) was measured at the mid-point of the left arm.

Following delivery of placenta a tissue sample was obtained by cutting a one cm cube of tissue from both the central and peripheral cotyledons. These were fixed in 10% neutral buffered formalin. Biopsies were embedded in paraffin wax, sliced into thin sections and stained with Giemsa and/or haematoxylin and eosin. Slides were examined at the Histopathology Department, College of Medicine, Blantyre, by two observers, with discrepant findings assessed by a pathologist. Cases were women with placental malaria, and controls those with no evidence of infection. Placental malaria was histologically classified as acute, based on the presence of parasites, chronic, based on the presence of parasites and haemozoin, and past infection if haemozoin alone was present. Active infection included both acute and chronic categories [14].

### Study procedures

A maternal peripheral blood sample was obtained after delivery and placed in a EDTA tube. Thick and thin blood smears were stained with Giemsa and screened for malaria parasites. A full blood count was performed by an automated Beckman Coulter AcT 8 S/N. AA322060 (http://www.coulter.com). Ferritin and sTfR were quantified by enzyme-linked immunoassays (http://www.omegadiagnostics.co.uk;http://www.oriondiagnostica.fi). Iron deficiency was defined as a sTfR:log ferritin ratio > 1.6, as this cut-off has been shown to best predict iron deficiency based on bone marrow iron stores in Malawi, an area with high infection pressure [15]. DNA analysis was used to identify maternal genetic polymorphisms: ABO blood group, sickle cell polymorphism (HbS), and glucose -6-phosphate dehydrogenase deficiency (G6PD). Polymerase chain reactions (PCRs) were analysed by restriction fragment length polymorphism technique using restriction enzymes NlaIII and DdeI for G6PD and HbS respectively. All RFLPs were electrophoresed for 45 minutes on 2.5% agarose gel stained with ethidium bromide and visualized with a UVP GelDoc - It imaging system, USA.

Maternal parity was included in the analysis as a known correlate of placental malaria risk. Adolescence was defined as < 20 years. Chi-square, Mann-Whitney and student t tests were used for comparisons using SPSS version 18. Multivariate logistic regression was used to describe factors associated with placental malaria.

## Results

Between February-June 2004 and January-July 2005 a total of 112 infected cases were identified who were compared with the first 110 women identified with no evidence of placental infection. Over 95% of women had received one or more doses of sulphadoxine-pyrimethamine as intermittent treatment (IPTp-SP). There were no significant differences between cases and controls for the following parameters: mean age and parity, proportion with low mid-upper arm circumference (< 23 cm), marital or literacy status, adolescents, frequency of use of insecticide treated bed nets or uptake of IPTp-SP during pregnancy (all p > 0.1). 44.1% of cases and 42.5% of controls were anaemic (Hb < 11g/dl), There were no differences between cases and controls in prevalence of severe anaemia (Hb < 8 g/dl, mean 5.9%), G6PD deficiency (mean 18.7%), or sickle cell trait (mean 44.5%), (all p > 0.3).

Blood group O phenotype was less frequent in multigravidae with active (35.4%, p = 0.037), or acute infection (34.8%, p = 0.03) than controls (54.8%), while the converse was true for primigravidae (61.5%, p = 0.06; and 63.2%, p = 0.09; versus 37.1%). Sera was available for iron biomarkers for 92 cases and 68 controls. Of cases 57.6% had acute, 9.8% chronic, and 32.6% past infection. Women with either acute (p = 0.001), or acute and chronic (p = 0.006) placental malaria were less likely to have iron deficiency than women without placental malaria infection (Table 1). For all infection categories (acute, chronic, past) the odds ratio for iron deficiency was 0.4 (95% CI 0.2-0.8, p = 0.01). Iron deficient multigravidae, but not primigravidae, were less likely to have placental malaria (all infection categories, p = 0.002) (Table 1).

**Table 1:** Risk of iron deficiency in cases and controls

		CASES		CONTROLS	
	
Iron Status	All infected	**Active **(acute+ chronic)	Acute Infection	Past Infection	No Infection
	n/N (%)	n/N (%)	n/N (%)	n/N (%)	n/N (%)
	OR (95% CI)	OR (95% CI)	OR (95% CI)	OR (95% CI)	
**Iron deficiency: primigravidae and multigravidae**	44/92 (47.8)	28/62 (45.2)	21/53 (39.6)	16/30 (53.3)	47/68 (69.1)
	0.4 (0.2-0.8)	0.4 (0.2-0.8)	0.3 (0.1-0.7)	0.5 (0.2-1.3)	
	p = 0.01	p = 0.006	p = 0.001	p = 0.13	

**Iron deficiency: primigravidae**	20/37 (54.1)	13/24 (54.2)	8/17 (47.1)	7/13 (53.8)	9/16 (56.3)
	0.9 (0.3-3.5)	0.9 (0.3-3.9)	0.7 (0.1-3.4)	0.9 (0.2-5.1)	
	p = 0.9	p = 0.9	p = 0.8	p = 0.9	

**Iron deficiency: multigravidae**	24/55 (43.6)	15/38 (39.5)	13/36 (36.1)	9/17 (52.9)	38/52 (73.1)
	0.3 (0.1-0.7)	0.2 (0.1-0.6)	0.2 (0.1-0.6)	0.4 (0.1-1.5)	
	p = 0.002	p = 0.002	p = 0.001	p = 0.1	

**N**	92	62	53	30	68

All infected = acute, chronic and past infection

OR: odds ratio for infected category versus non-infected

Three models were used for the regression analysis, each for a placental malaria category (all categories, active, or past). Factors in the model were those with P < 0.1 in univariate analysis. These were iron deficiency, parity, ABO phenotype and adolescence. Risk of iron deficiency was reduced for combined infection categories (p = 0.008), active infection (p = 0.007), or acute infection (p = 0.001)(Table 2). In multigravidae there was reduced risk of malaria infection for all infection categories (p=0.03). Active infection was increased in adolescents (p=0.04).

**Table 2:** Adjusted odds ratios for factors related to placental malaria

Factor	All infected		Active (acute + chronic)		Acute Infection	
	Odds ratio	p value	Odds ratio	p value	Odds ratio	p value
**Iron Deficiency**	0.40 (0.20-0.79)	0.008	0.36 (0.17-0.76)	0.007	0.29 (0.13-0.62)	0.001

**Multigravidae**	0.45 (0.22-0.92)	0.030	0.64 (0.26-1.58)	0.34	0.92 (0.35-2.41)	0.87

**Adolescence**	1.53 (0.63-3.72)	0.33	2.26 (1.01-5.07)	0.046	2.01 (0.85-4.72)	0.11

**Blood Group O**	0.91 (0.47-1.76)	0.78	0.74 (0.36-1.55)	0.43	0.71 (0.33-1.52)	0.38

Brackets: 95% confidence interval

Reference: no placental infection (Controls)

All infected: acute, chronic and past infection

## Discussion

Iron deficiency was less frequent in women with evidence of acute or chronic placental malarial infection indicating that women with placental infection have better iron status than those without. There was no difference in use of IPTp-SP during pregnancy between cases and controls, and the association remained highly significant controlling for maternal ABO phenotype.

The importance of these results relates to the influence which maternal iron status has on parity specific malaria immunity, especially as the results were most significant for multigravidae. The cumulative iron requirements of successive pregnancies would increase prevalence of iron deficiency leading to reduced risk for placental malaria in multigravidae. As there is a need to supplement these women with iron this is likely to increase malaria risk despite acquisition of parity specific malaria immunity. These results support the findings from a study in Tanzania based on intervillous blood smears although that study reported malaria was less prevalent among women with iron deficiency especially during the first pregnancy [9]. It will be important to establish the relative importance of differences in iron effects between parity groups in a much larger study in order to determine confounding effects on parity specific malarial immunity, and in relation to differential effects on malaria risk of iron supplementation in pregnancy.

The calculation of body iron store based on the sTfR:SF ratio provides the best estimate of iron status in adult men and non-pregnant women in populations where infectious disorders are not prevalent. Its utility where women are living under endemic conditions for malaria is less clear and in severely anaemic HIV infected adults had low sensitivity based on bone marrow iron stores [16]. It has good sensitivity and specificity (> 70%) for reduced bone marrow iron stores in Malawian children with severe anaemia (Hb < 5g/dl) irrespective of the presence of infection [15]. The prevalence of iron deficiency in all women was not high (47.8%), although similar to a previous estimate from this area [17]. Other causes for anaemia are likely to be important including vitamin A and B12 deficiencies.

Four hypotheses might explain these effects of iron status in the context of malaria. These are: the availability of non-transferrin bound iron which might influence the growth of the malaria parasite which may be an acute effect related to recent iron intake or supplementation; an effect on the oxidant/anti-oxidant balance and oxidative free radical reactions in the placental intervillous blood which may alter cellular immune function and macrophage iron metabolism; altered expression of vascular endothelial molecules which may influence adhesion and sequestration of *P.falciparum *parasites; and altered erythropoiesis with down regulation of parasite invasion of young red cells.

All these women received haematinics of iron (60 mg) and folic acid (5 mg) in short daily courses mostly in the second half of pregnancy. Women were not routinely screened for anaemia at first antenatal attendance or during pregnancy and there was no preferential prescription of haematinics for selected women with anaemia. This is important because women with malarial anaemia could receive therapeutic iron supplements for anaemia treatment, reducing iron deficiency, and then independent of their iron status, these same women may be more likely to have placental malaria due to a higher malaria exposure risk. Although the possibility that some women received additional iron treatments cannot be excluded, the number was nevertheless likely to be small and the policy was to uniformly prescribe haematinics to all women.

This study needs to be replicated in women living under different levels of malaria transmission in order to determine the magnitude of these effects in subjects with different levels of malaria immunity. The small sample size and lack of available sera from all subjects limited detailed sub-analyses, for example, placental malaria iron interactions in younger adolescents less than 16 years. A large multi-centre case control study is required to adequately address this.

As many millions of pregnant women are exposed to malaria annually there is a priority to establish if reversing such iron deficiency states through routine supplementation programs either prior to or during pregnancy enhances malaria risk especially in areas where malaria control and surveillance is limited. It is particularly important to assess this in adolescents as their malaria immunity is lower than that in older women and many will experience pregnancy while still adolescent.

## Competing interests

The authors declare that they have no competing interests.

## Authors' contributions

ES carried out the field study, participated in the analysis and preparation of the manuscript; GH co-ordinated and participated in the laboratory analyses; GK supported the data preparation and analysis; PK participated in the study design and co-ordination and helped to draft the manuscript; BB conceived the study and participated in its design, co-ordination, analysis and helped draft the manuscript. All authors have read and approved the final manuscript.
